# Progress in the development of a fully implantable brain–computer interface: the potential of sensing-enabled neurostimulators

**DOI:** 10.1093/nsr/nwac099

**Published:** 2022-05-24

**Authors:** Yue Chen, Guokun Zhang, Linxiao Guan, Chen Gong, Bozhi Ma, Hongwei Hao, Luming Li

**Affiliations:** National Engineering Research Center of Neuromodulation, School of Aerospace Engineering, Tsinghua University, China; National Engineering Research Center of Neuromodulation, School of Aerospace Engineering, Tsinghua University, China; National Engineering Research Center of Neuromodulation, School of Aerospace Engineering, Tsinghua University, China; National Engineering Research Center of Neuromodulation, School of Aerospace Engineering, Tsinghua University, China; National Engineering Research Center of Neuromodulation, School of Aerospace Engineering, Tsinghua University, China; National Engineering Research Center of Neuromodulation, School of Aerospace Engineering, Tsinghua University, China; National Engineering Research Center of Neuromodulation, School of Aerospace Engineering, Tsinghua University, China; Precision Medicine & Healthcare Research Center, Tsinghua-Berkeley Shenzhen Institute, Tsinghua University, China; IDG /McGovern Institute for Brain Research at Tsinghua University, China; Institute of Epilepsy, Beijing Institute for Brain Disorders, China

## Abstract

This perspective article investigates the performance of using a sensing-enabled neurostimulator as a motor brain-computer interface.

With the advances in neurophysiological recording technology and active implantable medical device development, sensing-enabled neurostimulators have become an emerging technology of the fully implantable brain–computer interface (BCI). These implantable neurostimulators are designed for deep brain stimulation (DBS), a well-established treatment for movement disorders and a promising treatment for psychiatric disorders. By integrating neural recording, sensing-enabled neurostimulators can sample local field potentials (LFPs) through the available contacts of the DBS electrode while delivering stimulation.

In the past decade, fully implantable BCIs have been developed considerably by integrating low-power recording, inductive recharging and wireless communication [1]. These implantable BCIs have the potential to reveal precise brain states and human behaviors in complex conditions by recording neural activities with a high spatial and temporal resolution [1,2]. Currently, fully implantable BCIs are designed bi-directionally, i.e. for decoding brain activities and modulating brain networks [1]. The latter modulation effects have become an essential aspect of BCIs [3]. However, most of the fully implantable BCIs have only been investigated in preclinical animal models [1,2]. From the currently available solutions, sensing-enabled neurostimulators are particularly striking when it comes to achieving fully implantable BCI applications. The functionality of recording invasive neural activities after implantation would allow us to investigate fully implanted BCIs in a clinical setting. The measured brain signals could be used as inputs in classical BCI systems, as biomarkers for the clinical assessments, and as triggers and feedback for modulating stimulation pulses [4]. Previous studies have validated the feasibility of using a sensing-enabled neurostimulator as a fully implanted BCI. In Mariska Vansteensel's study, a neurostimulator was used to sense the LFPs in the motor cortex of a patient with late-stage amyotrophic lateral sclerosis and transmit the signals to a real-time brain-control typing system [5]. More and more clinical researchers are trying to validate the feasibility and the effectiveness of using the sensing-enabled neurostimulator for long-term closed-loop stimulation [6,7].

In our previous studies, we designed a sensing-enabled neurostimulator for longitudinal brain signal recording in clinical practice [8]. Recently, we improved the neurostimulator system and investigated its potential as a motor BCI. As shown in Fig. 1a, the sensing-enabled neurostimulator is equipped with Bluetooth communication capability. Eight LFP channels (24-bit resolution) could be differentiated between pairs of contacts and transmitted synchronously to a recording computer or a mobile phone.

**Figure 1.**
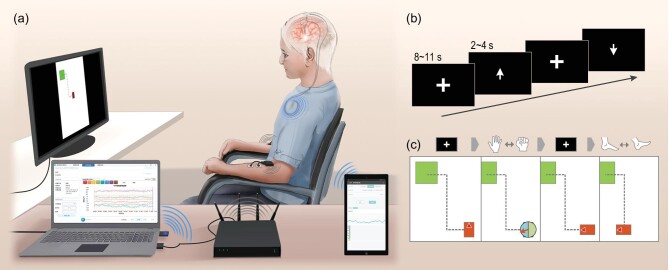
Illustration of the fully implantable brain–computer interface. (a) The data recording and decoding system. The sensing-enabled neurostimulator recorded and transmitted local field potentials (LFPs) to the computer via Bluetooth communication (the blue coils). It could also transmit LFPs to a mobile phone via Bluetooth communication. A screen was placed 60 centimeters before the patient to show the instructions or interactive interfaces. During movement-related experiments, the patient followed the instruction on the screen and performed voluntary movements. For the study of the patterns of movement-related LFPs, electromyography and acceleration recordings were wirelessly transmitted to the computer and synchronized with LFP recordings. (b) The paradigm of movement-related LFP recording. Each trial began with a resting interval of random duration between 8 and 11 s. During this period, a fixed cross was shown in the center of the screen and the patient was asked to keep their focus on it. Then an arrow replaced the cross and instructed the patient to perform continued upper limb movements (repeated bilateral hand closing and opening) or lower limb movements (repeated bilateral instep extension and flexion) for a random duration between 2 and 4 s. (c) The interface of the motor BCI based on movement-related LFPs. The white area is an enclosed area of 19.08 cm × 25.44 cm, which simulated an area of 9 m × 12 m. The red block is the simulative wheelchair. The green block represents the target area. The dashed line suggested a recommended path to hit the target area. When the decoder detected continued upper limb movements, a dial with a rotary pointer would appear to select turning left or right. When the decoder detected lower limb movements, the red block would move forward along the current direction. When the classifier detected a resting state, the block would stop.

The transmission delay is <10 ms, and the maximum sampling rate is 1 kHz. The wireless communication distance is ∼2 m, and the rechargeable battery life is 10 years. This stimulator could continuously sample and transmit LFPs for ∼6 to 12 hours, depending on the number of the synchronous channels.

To validate the safety and functionality of the sensing-enabled neurostimulator, a patient with Parkinson's disease was recruited for DBS surgery. The study procedures were approved by the ethics committees of Beijing Tiantan Hospital of Capital Medical University, and informed consent was obtained. Implantation surgery was performed by neurosurgeons using a standard procedure with frame-based stereotaxic and microelectrode recording techniques. The DBS electrodes were implanted in the bilateral subthalamic nucleus (STN). The post-operative improvement was 84.21% (relative decrease of UPDRS-III scores under the stimulation-on state to the stimulation-off state, after washing in/out for at least 30 minutes). The post-operative improvement confirmed the locations of the electrodes and the essential stimulation safety and functionality of the neurostimulator.

To validate the functionality of the chronic LFP-sensing and real-time data transmission of the neurostimulator, LFP-based BCI experiments were conducted after 14 months of DBS implantation. As shown in Fig. 1a, LFPs were recorded during DBS-off state via Bluetooth communication. Four LFP channels in each STN (eight channels in total) were sampled and the sampling rate was 500 Hz. To record the neural activities during the onset of voluntary movement, surface electromyography (EMG) and accelerometers were wirelessly recorded by a commercial amplifier (Noraxon USA Inc., Scottsdale, AZ, USA). The surface EMG electrodes were placed on the bilateral upper forearm and lower leg muscles, and the accelerometers were placed on the bilateral hands and instep. A screen was placed 60 cm in front of the patient to show movement instructions. The instructions were generated by the instructions system in the computer. The patient was seated in an armchair and instructed to perform voluntary movements. For multimode signal synchronization, a spare surface EMG channel was used to detect the DBS artifacts in the LFP channels, and another spare EMG channel was used to detect a serial port level outputted by the instruction system. Figure 1b shows the paradigm of movement-related LFP recording. In each trial, the patient performed continued upper limb movements (bilateral hand closing and opening) or lower limb movements (bilateral instep extension and flexion) for a random 2–4 s. The resting interval was random 8–11 s. The trials of upper and lower limb movements were counterbalanced.

To decode the movement-related LFP patterns, we developed a machine-learning classifier based on LFP features in the frequency domain. Previous studies revealed that the STN-LFP beta oscillation is modulated by voluntary movement. A previous study illustrated that online decoding beta suppression as a neurofeedback signal linked the subthalamic oscillations and the motor impairment of DBS patients [9]. By extracting the beta oscillations during voluntary movements, it is possible to predict movement onset and classify upper and lower limb movements [10–13]. In our study, features in the alpha band (10–13 Hz), the beta band (13–35 Hz) and the gamma band (35–50 Hz) were used for movement-states decoding. To simulate real-time testing, the features were extracted for every 100 ms data segment. To improve the frequency estimation performance, we expanded the 100 ms window in each end to construct a smooth window of 600 ms (400 ms before and 100 ms after the current window). The data were first filtered by a 6th-order 6–60 Hz Butterworth filter and the frequency domain features were extracted using the wavelet transform (morlet) with a frequency resolution of 1 Hz. The machine-learning classifier was a one-hidden-layer recurrent neural network with 5 input neurons and 25 hidden neurons. The classifier's inputs were the frequency domain features, and the outputs were the labels of the three movement states (resting state, upper limb movements and lower limb movements). For training, 100 trials of LFP recording were used, and 20 trials were used for prediction. In the offline prediction, the sensitivity of each category was 95.33% (resting state), 70.31% (upper limb movement) and 78.96% (lower limb movement). The selectivity of each category was 91.36% (resting state), 78.44% (upper limb movement) and 92.42% (lower limb movement). The general accuracy of prediction was 89.85%. The decoding performance was comparable with previous studies. In reference [10], the researchers predicted the onset of movements with 95% sensitivity and 77% specificity. In reference [11], the accuracy of movement decoding achieved 99.6% and the accuracy of laterality classification reached 77.9%. In one study, the low-frequency bursts were used to predict the intended movements, and the area under the receiver operator characteristic curve was ∼0.80 [13]. Another study decoded the movement states in stepping cycles based on subthalamic LFP frequency features and the accuracy was above 81.3% [12].

To evaluate the performance of the motor BCI based on the classifier, we designed a two-dimensional center-out task to simulate a wheelchair control. As shown in Fig. 1c, at the beginning of each trial, a red block (simulative wheelchair) randomly appeared in the white area (19.08 cm × 25.44 cm, simulating an area of 9 m × 12 m). The patient freely performed the three movements to control the red block. During online testing, the feature extraction process was the same as the offline analysis, and the control signal was updated every 100 ms. When the classifier detected five consecutive samples of upper limb movements, a dial with a rotary pointer would appear to select the direction. The block would then turn 90 degrees to its left or right. When the classifier detected a sample of lower limb movements, the block would start to move forward along the current direction with an initial speed of 0.32 cm/s (simulating typical wheelchair initial speed 0.15 m/s). The distance the block moved would be 0.032 cm. If the classifier detected continued lower limb movements, the block would speed up with an acceleration of 1.06 cm/s^2^ (typical wheelchair acceleration 0.50 m/s^2^). The maximum speed was 2.12 cm/s (typical wheelchair maximum speed 1.00 m/s). When the classifier detected a sample of the resting state, the block would stop moving. A valid trial ended when the block hit the green target area. After transitory training, the patient successfully completed 16 of 21 trials (76%). The average time to hit the target area was 63.25 ± 29.67 s. The results confirmed the feasibility of using the sensing-enabled neurostimulator to control a two-dimensional block.

In conclusion, we investigated the performance of a sensing-enabled neurostimulator when used as a motor BCI. To our knowledge, this is the first trial demonstrating the potential for a fully implantable BCI based on motor information in STN-LFP. It should be noted that the fully implantable BCI in the current study is compromised since all the computations were implemented in an external computer, and the decoding was performed based on actual movements. Future studies could investigate the detection of various behavioral and physiological states, such as imaginary movements, sleep patterns and mental states, and explore the possibility of on-board computations in the sensing-enabled neurostimulator. Standing at the intersection of neuromodulation and neural decoding, sensing-enabled neurostimulators have opened a window onto the deep brain and could largely propel the development of closed-loop BCIs for clinical applications.
